# A review of aeroelastic instabilities and resonance effects in wind turbine blade dynamics

**DOI:** 10.1177/0309524X251405726

**Published:** 2025-12-03

**Authors:** Muhammad Usman Saram, Jianming Yang

**Affiliations:** 1 Faculty of Engineering and Applied Science, 7512 Memorial University of Newfoundland, St John’s, NL, Canada

**Keywords:** wind turbine, blade design, blade loads, aeroelastic instabilities, resonance

## Abstract

Over the years, wind turbine blades have become increasingly larger and more flexible to achieve higher efficiency and lower energy costs, which brings more issues related to forced resonance and aeroelastic instabilities because of higher dynamic loads and complex inflow conditions. The manuscript systematically reviews the literature, covering experimental versus computational studies, reduced-order models (ROM), machine learning (MI) based aeroelastic models, Euler-Bernoulli beam models, Timoshenko beam models, geometrically exact beam formulations, and computational fluid dynamics (CFD) models coupled with structural dynamics models to investigate primary and internal resonances and dynamic stalls for National Renewable Energy Laboratory (NREL) 5-MW and International Energy Agency (IEA) 15-MW wind turbines. The article also addresses vibration mitigation techniques, including passive, active, and semi-active control, to resolve aeroelastic instabilities. Most studies have assumed linear aeroelastic models and isotropic blade material for initial structural dynamics analysis. The higher mode frequencies computed using the Euler-Bernoulli model differ by approximately 5.23%, those using the Timoshenko model by 3.13%, and those through the Rayleigh model by 3.4% from the geometrically exact formulations employed. Euler-Bernoulli models significantly overestimated flutter speeds compared to the geometrically exact beam model for the NREL 5-MW blades. A key takeaway is that modern, prolonged, flexible blades are sensitive to flutter instabilities, where aerodynamic damping can drop significantly at certain operational speeds. The Euler-Bernoulli beam model proved to be a valuable tool at the initial design stage due to its simplicity and computational efficiency. Future research on managing forced resonance and dynamic stalls in ultra-large blades should focus on integrating nonlinear modeling, cutting-edge materials and structures, artificial intelligence (AI)-powered digital twins, and exploring targeted active control techniques.

## Introduction

For thousands of years, people have utilized wind energy for various purposes, including navigation on the oceans, timber harvesting, oil extraction, grain milling, tobacco processing, and water pumping. Simpler wind devices have been used for thousands of years, but significant advancements in wind energy technology occurred centuries ago. Around 200 BC, vertical-axis windmills emerged along the Persian-Afghan borders, whereas horizontal-axis windmills appeared much later in the Netherlands and Mediterranean regions between 1300 and 1875 AD (John and Zafirakis, 2011; Musgrove, 2010). The initial contemporary horizontal-axis wind turbines (HAWTs) were engineered explicitly for electricity generation and constructed in Denmark to address the need for rural electrification (Gipe and Mollerstrom, 2022). Its diameter is about 12.5 m, using five or six variable-pitch blades to drive a 40-kW asynchronous generator (Gipe and Mollerstrom, 2022).

Safety concerns of nuclear power and sharp rises in oil prices since the 1970s ultimately led certain politicians to recognize the limited nature of the world’s fossil fuel reserves. Consequently, this awareness prompted more progressive governments to allocate resources towards initiatives utilizing renewable energy (Fleming and Probert, 1984).

Wind energy is a significant option among renewable energy sources due to its environmental benefits and widespread availability. Global wind power installations reached a record level in 2023, accounting for 30% of total renewable energy in power generation. China remains the primary contributor to the growth of wind power, accounting for roughly 66% of the global capacity increase recorded last year (Neufeld, 2024). In the United States, wind energy capacity has more than doubled over the past decade. As of 2023, Canada has a total wind power installation of 16,989 MW, with an annual growth rate of 8.1% between 2013 and 2023 (Davenport and Wayth, 2024). Globally, wind energy is experiencing robust growth, particularly in Europe, where countries such as Germany, Spain, Sweden, and the United Kingdom are among the prominent producers (Davenport and Wayth, 2024).

The recent trend in wind turbine design is to scale up with lighter and more flexible blades, resulting in significant deformations within the system and making nonlinear effects more pronounced. These effects include problems related to aeroelastic instability and resonances. Severe structural and aerodynamic coupling may lead to negatively damped modes, contributing to aeroelastic instability problems and significantly impacting the turbine’s lifespan. Coincidence of the natural frequencies of various blade modes with excitation frequencies leads to resonance, including primary, subharmonic, and superharmonic resonances, as well as multi-valued resonances, which reduce the system’s efficiency and potentially cause instability.

This review is organized as follows. Section 1 (Introduction) provides an overview of recent trends in wind turbine blade size and wind energy capacity. Section 2 (Materials and methods) outlines the primary sources used in this study, presenting the selection procedure through a PRISMA flow chart. Section 3 (Wind turbine blade failures) identifies the global failures of wind turbines due to blade damage and highlights the primary sources of such damage. Section 4 (Wind turbine blade—mode shapes) provides a basic introduction to the mode shapes of a wind turbine blade. Section 5 (Nonlinear dynamics and resonance of wind turbine blades) examines previous research on the dynamics of wind turbine blades, with an emphasis on beam models for solving nonlinear equations, the occurrence of resonances due to nonlinearities, and the blade’s vibrations considering both bending and torsion modes. This section also highlights the shortcomings of the existing literature for future research on ultra-long blades. Section 6 (Aeroelastic instabilities) highlights prior studies on aeroelastic instabilities, such as classical flutter and stall-induced vibrations (SIV) in wind turbine blades, while also identifying research deficiencies in the current literature. Vibration control techniques, including passive, active, and semi-active control, are discussed in Section 7 (Vibration mitigation strategies) to resolve aeroelastic instabilities. Section 8 (Future directions) mentions emerging trends and future research directions. The outcomes and conclusions are summarized in Section 9 (Conclusions).

## Materials and methods

**Figure 1.**
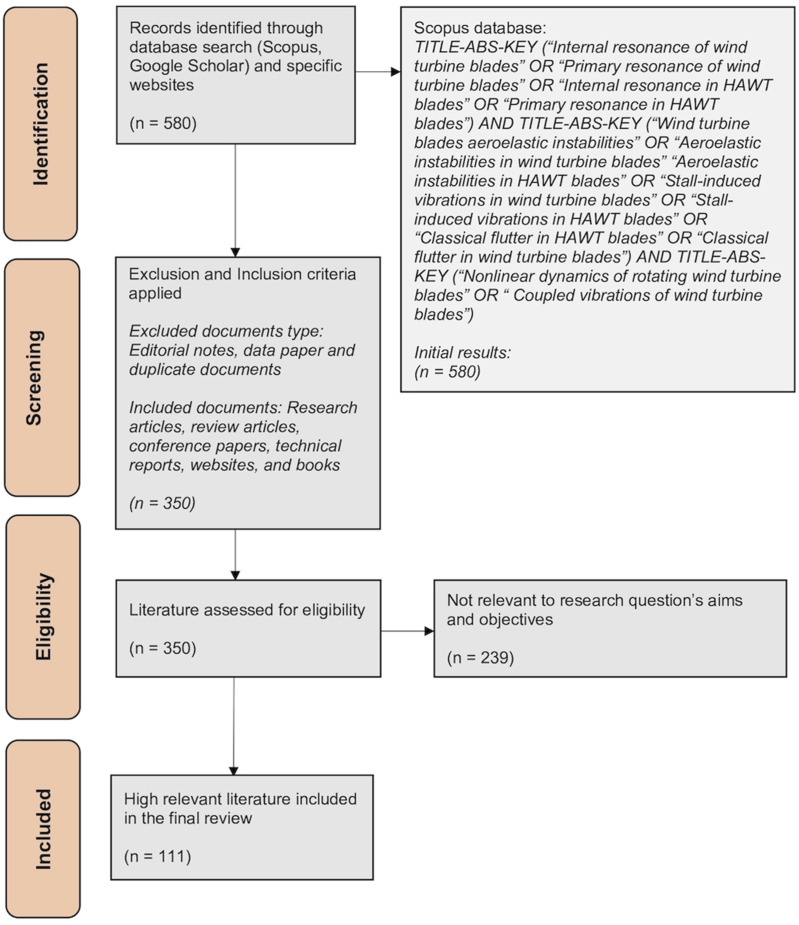
PRISMA flow diagram: identification and screening of studies for the systematic review of aeroelastic instabilities and resonance effects in wind turbine blade dynamics.

The data collection procedure was conducted using a PRISMA-based flowchart, structured into four stages: identification, screening, eligibility, and inclusion, as illustrated in Figure 1. A query was run in the Scopus database using advanced search in the Identification phase. The search contained the following advanced query: TITLE-ABS-KEY (“Internal resonance of wind turbine blades” OR “Primary resonance of wind turbine blades” OR “Internal resonance in HAWT blades” OR “Primary resonance in HAWT blades”) AND TITLE-ABS-KEY (“Wind turbine blades aeroelastic instabilities” OR “Aeroelastic instabilities in wind turbine blades” “Aeroelastic instabilities in HAWT blade” OR “Stall-induced vibrations in wind turbine blades” OR “Stall-induced vibrations in HAWT blades” OR “Classical flutter in HAWT blades” OR “Classical flutter in wind turbine blades”) AND TITLE-ABS-KEY (“Nonlinear dynamics of rotating wind turbine blades” OR “Coupled vibrations of wind turbine blades”). This resulted in an initial set of 580 documents. In the screening phase, the inclusion and exclusion criteria were used. The document types, including research articles, review articles, conference papers, technical reports, websites, and books, published between 1945 and 2025, were retained. Editorial notes, data papers, and duplicate documents were excluded to maintain the consistency and relevance of the dataset, and the dataset was refined to 350 documents. During Eligibility phase, technical literature was assessed for alignment with the research objectives, focusing on resonance phenomena and aeroelastic instabilities in wind turbine blades. Finally, in the Inclusion stage, the resulting dataset of 111 high-relevance documents was confirmed for use in the final review.

The unique characteristic of this paper, in comparison to other papers, is multifold. Most researchers have reviewed the failure modes, structural controls, and vibration issues, as well as technology trends of the wind turbine system; however, they have not provided a comprehensive review of the individual components, particularly the wind turbine blades. Secondly, the negative aerodynamic damping (aeroelastic instability) can lead to large oscillations when the magnitude of the negative aerodynamic damping exceeds that of the structural damping. Therefore, merely preventing the coincidence of natural frequencies of various modes with excitation frequencies is insufficient. It is crucial to avoid both resonance and aeroelastic instabilities to achieve an aeroelastically stable blade design; therefore, this paper will thoroughly review these topics. Furthermore, this study will identify research gaps and areas of resonance and aeroelastic instabilities that have been neglected by prior studies, aiming to encourage academics and industry professionals to explore these areas further, ultimately contributing to the future design of ultra-large blades. These blades are more likely to exhibit substantial nonlinearities because of their length and flexibility.

## Wind turbine blade failures

**Figure 2.**
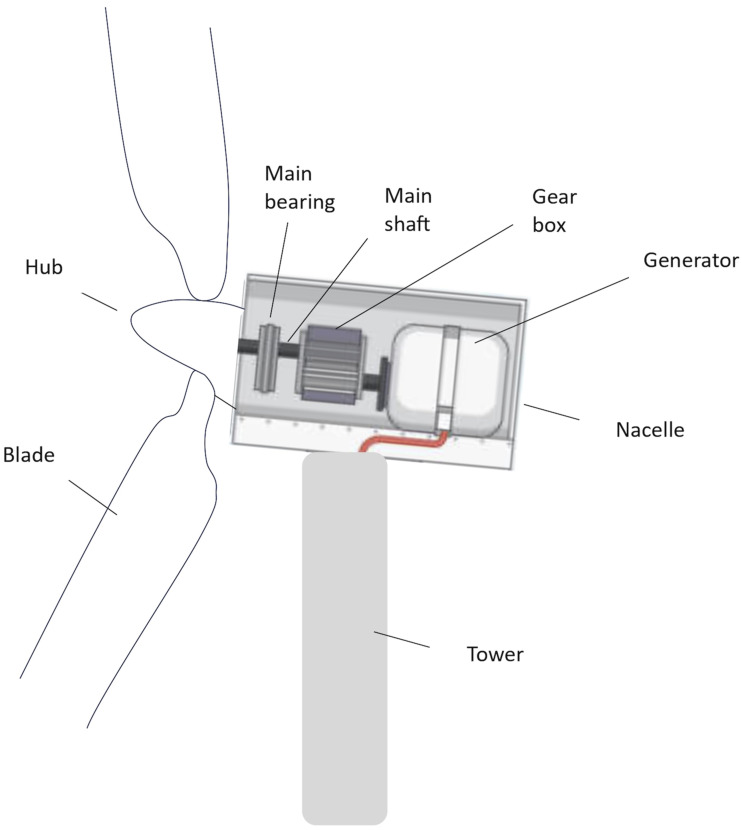
Schematic of a horizontal axis wind turbine and its major components.

Wind turbines comprise over 8000 individual components, which can be further classified into six main categories. Blades, nacelle, and tower are critical for the operational capabilities of a wind turbine, as illustrated in Figure 2. The tower is anchored to a concrete foundation supporting the blades and nacelle. The nacelle depicted in Figure 2 serves as the core component of the turbine. It contains the drive train, gearbox, and the generator. The size of the nacelle may vary depending on the turbine’s classification, which is categorized as small, medium, or large; however, it is typically designed to occupy a compact space, facilitating maintenance activities.

**Figure 3.**
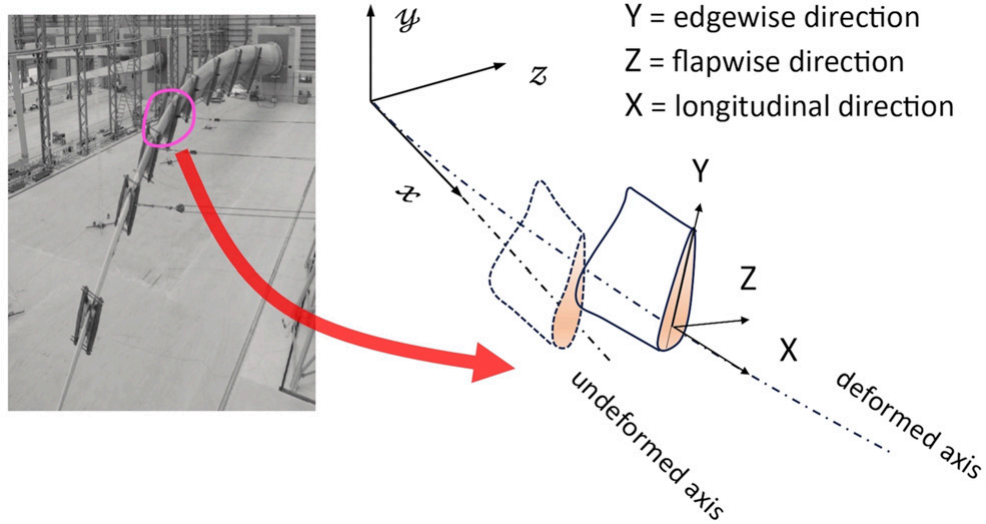
Wind turbine blade deformation: deformed and undeformed configurations.

A blade consists of several components: the root, chord, mid-span, and tip. Each of these elements plays a crucial role in generating uplift. The turbine tip facilitates the formation of a helical vortex. Typically, turbine roots are designed to withstand various stresses from fluctuating wind loads over time (Watson and Serrano, 2010). While mid-span controls bending and shear loads, which are transferred from mid-span to the root, the chord produces lift. The pressure and suction surfaces are interconnected by the shear webs that join the upper and lower sections of the blade (Mishnaevsky et al., 2017). Globally, the three-blade horizontal-axis design of wind turbines is the most prevalent. This configuration is favored for its efficiency, acceptable noise levels, and longevity. Although various alternative designs have been investigated, they have primarily been rejected due to higher costs and reduced efficiency. A typical individual blade of a three-blade HAWT is shown in Figure 3.

Wind turbines represent intricate machinery, comprising numerous technologies that operate under challenging environmental and operational conditions. These include variable loads caused by gusty winds and challenges posed by humidity, dust, fatigue, and a broad range of temperatures (Chen, 2018; Kong et al., 2022; Li et al., 2015; Mishnaevsky, 2019). Such harsh conditions may lead to an increase in component failures and operational issues. Within this integrated system, certain parts are more critical than others. Wind turbine blades often operate in these conditions, which can lead to blade deformation and delamination (Sundaresan et al., 1999).

**Figure 4.**
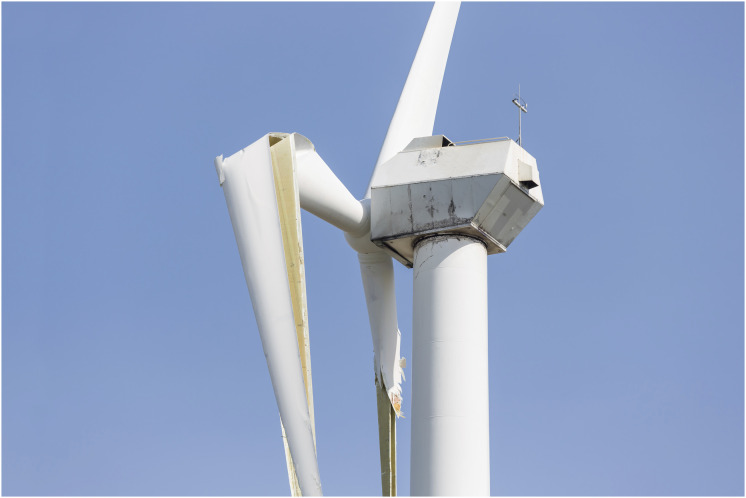
Damaged wind turbine in the southwest of Nantucket Island, Massachusetts, USA, showing a single broken blade.

According to a study conducted over 5 years involving 350 operational wind turbines across Europe, Carroll et al. (2016) indicated that the components most prone to failure are blades, gearboxes, and generators. An estimated 700,000 blades operate worldwide, resulting in an average of 3800 blade failure incidents annually. The total repair budgets in Europe increased from USD 4.7 million to USD 8.6 million between 2019 and 2020. Moreover, unplanned repairs saw a modest 2% increase in Europe and a more significant 10% rise in America (Ziegler et al., 2018). Figure 4 illustrates a wind turbine that has a broken blade. Based on the wind turbine statistics from 2010 to 2012, blade fractures were the most common type of blade failure observed in China. These failures were primarily related to the deformation caused by prolonged operation, manufacturing defects, and blade vibrations (Lin et al., 2016).

**Figure 5.**
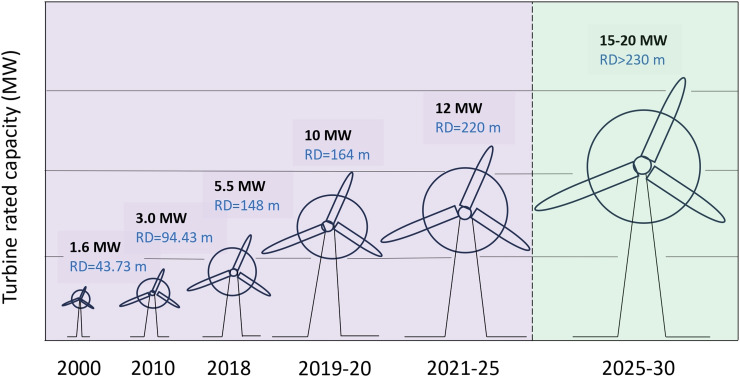
Blade size and power output of existing and planned offshore wind turbines (IRENA, 2019).

Based on Figure 5 (IRENA, 2019), the new strategy involves the production of taller and lighter blades aimed at maximizing wind energy capture. However, the increased flexibility may intensify vibration issues, making the blades more vulnerable to dynamic loads. Resonance occurs when the excitation frequency approaches one of the system’s natural frequencies. Furthermore, various cases of resonance may occur, leading to increased noise levels, reduced fatigue life, or potentially catastrophic blade failure. Aerodynamic coupling may lead to a negatively damped blade mode, which can contribute to aeroelastic instability issues such as stall and classical flutter, and substantially affect the turbine’s operation.

The recent trend towards lightweight wind turbine blades has led to a slender design, and these structures are more likely to undergo regular and significant deformations due to their exposure to harsh environmental conditions throughout their lifespan. Therefore, their reliability and structural integrity are critical from an economic and a safety standpoint. A newly developed rotor blade design must be certified before it can operate. The International Electrotechnical Commission (IEC) and Det Norske Veritas (DNV) have been instrumental in establishing design standards for wind turbine rotor blades, such as those outlined in DNV (2024), IEC (2019), and IEC (2020).

The design, structural components, and materials of wind turbine blades are strongly influenced by the mechanical loads acting on them, which are primarily due to inertial and aerodynamic loads. Due to the high aerodynamic efficiency of the wind turbine blade aerofoil, drag is considerably lower than lift. The turbine may experience varying wind intensities depending on the installation site. The IEC 61400-1 standard defines Class I turbines as those designed against higher average wind speeds and single gust intensities (IEC, 2019).

For larger wind turbines with diameters exceeding 70 m, such as the NREL 5-MW and IEA 15-MW, inertia loads resulting from the rotation and weight of the blades can be quite severe and should be considered. As shown in Figure 3, the primary forces determining the structural design of the blades are the flapwise and the edgewise bending moments. Furthermore, because of the higher magnitude of the lift force than the drag and gravity, the flapwise bending is a far more severe load than the edgewise bending. Per the IEC 61400-5 standard, the ultimate strength of the blade is verified by applying extreme loads that simulate worst-case scenarios by amplifying characteristic loads with specified safety factors (IEC, 2020). The loads are applied to simulate flapwise and edgewise bending moments, and the blade must withstand the ultimate design load without experiencing structural failure for a minimum hold time.

Fatigue loading results from gravitational cyclic loads and variations in wind intensity, humidity, and temperature, which equal the number of rotations throughout the turbine’s lifetime. Although most of these cycles have relatively small amplitudes, their amount in a wind turbine’s operational life, typically 20–25 years, makes them crucial. Therefore, IEC 61400-5 (IEC, 2020) and DNV-ST-0376 (DNV, 2024) certifications require fatigue analysis and testing to validate the blade’s durability over its lifetime by simulating millions of cyclic loads equivalent to the spectrum of loads that the blade will experience in real operation. The blade must withstand the required number of cycles in both flapwise and edgewise directions without initiating cracks or delamination.

## Wind turbine blade—mode shapes

**Figure 6.**
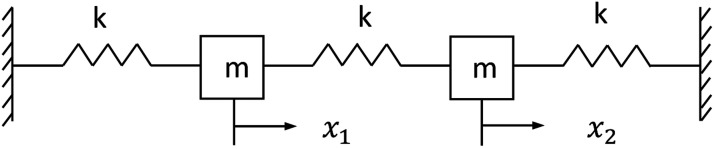
Graphical representation of a typical two-mass and three-spring system.

An elastic structure will vibrate in distinctive geometric shapes in response to periodic driving functions (Spera, 1994). These patterns are known as the structure’s eigenmodes or mode shapes, and each mode has a corresponding natural frequency. A simple two-mass and three-spring system is shown in Figure 6 to explain the eigenmodes. The natural frequencies of this system are given by:

(1) $$\omega_{1}=\sqrt{\frac{k}{m}},\omega_{2}=\sqrt{\frac{3k}{m}}$$

where m is the mass of each body, and k is the stiffness of each spring attached in the system of Figure 6. Eigenmodes corresponding to each natural frequency are given as:

(2) $$\left(\begin{array}{l} x_{1}\\ x_{2} \end{array}\right)=A_{1}\left(\begin{array}{l} 1\\ 1 \end{array}\right)\cos\left(\omega_{1}t+\psi_{1}\right)$$

(3) $$\left(\begin{array}{l} x_{1}\\ x_{2} \end{array}\right)=A_{1}\left(\begin{array}{l} \;1\\ -1 \end{array}\right)\cos\left(\omega_{2}t+\psi_{2}\right)$$

**Figure 7.**
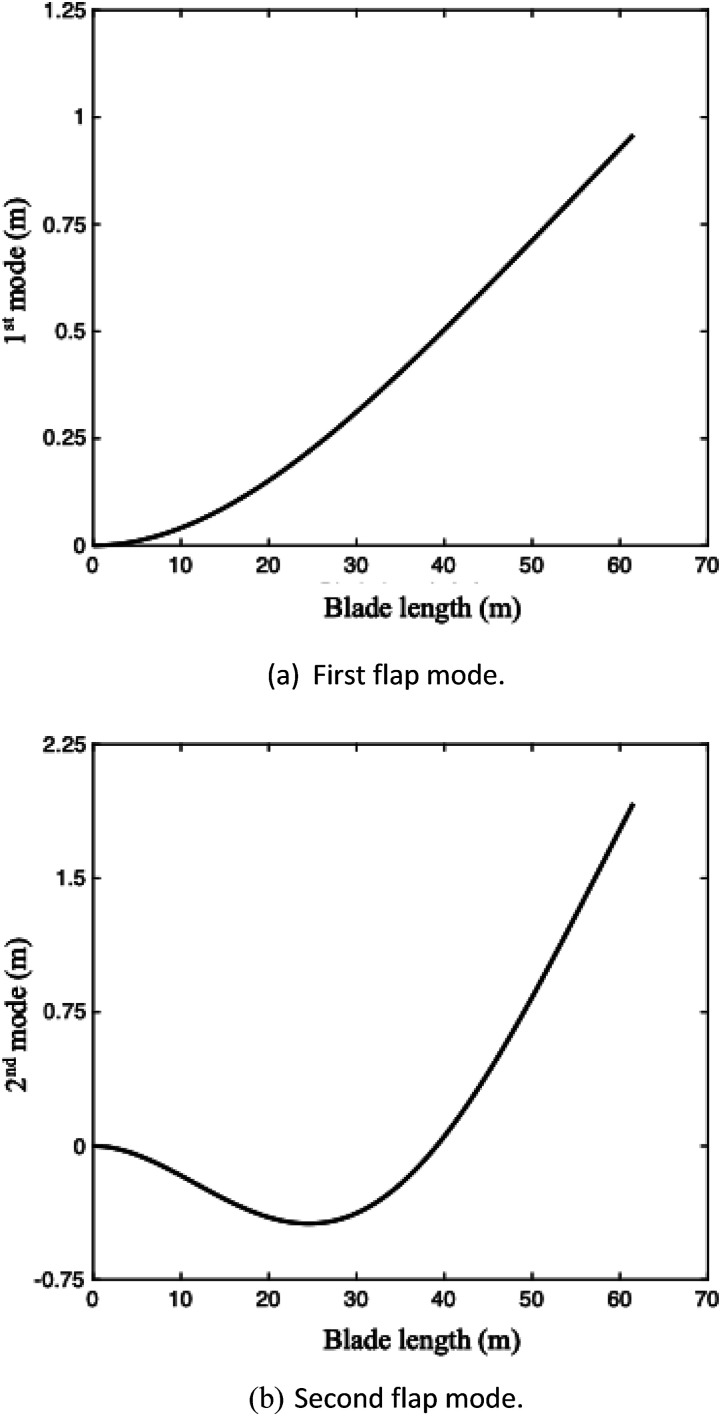
First two flap modes of the national renewable energy laboratory (NREL) 5-MW wind turbine blade having a blade length of 61.5 m.

Equation (2) for the first eigenmode demonstrates that both masses are moved in the same direction over an equal distance. In contrast, the second mode in equation (3) displays that the two masses move in opposite directions, again covering equal distances. A phase difference may arise, as represented by $\psi_{1}$ and $\psi_{2}$ in equations (2) and (3), when the excitation has a velocity. An infinite number of natural frequencies and eigenmodes exist for a continuous beam like the blade of a wind turbine. The first edge mode and the first two flap modes are the governing modes for the wind turbine blade, as explained by previous researchers (Jonkman et al., 2009). The first two flapwise mode shapes of the NREL 5-MW wind turbine blade are depicted in Figure 7. The first flapwise mode shows a smooth cantilever-like bending without crossing nodes (see Figure 7(a)). The maximum displacement occurs at the blade tip, diminishing linearly towards the fixed end. It is a purely out-of-plane mode with minimum mode coupling effects. It is dominated by the flexibility of the outer blade regions, making it extremely sensitive to aerodynamic and gravity forces. It will undergo a large amplitude of vibrations under these forces. In the second flapwise mode, the blade root and tip deflect in opposite directions with some crossing nodes, and there is higher curvature close to the mid-span (see Figure 7(b)). This indicates that it is not purely an out-of-plane (flapwise) mode, but instead has some in-plane (edgewise) motions. In other words, it is influenced most by the edgewise-flapwise coupling and will undergo a lower amplitude of vibrations compared to the first flapwise mode. Therefore, wind turbine blade mode shapes are necessary to investigate the nonlinear vibrations and instabilities of rotating blades, as described in the following Sections.

## Nonlinear dynamics and resonance of wind turbine blades

The wind turbine represents a distinct category of rotor system characterized by a long and heavy blade that rotates within a vertical plane, influenced by gravitational forces. It is well established that wind force intensifies with height above the ground surface, leading to periodic excitation of the blade as it rotates. Furthermore, there are instances when the wind is not perpendicular to the plane of rotation of the blade, resulting in additional periodic excitation forces acting on the blade, which creates an imbalance effect on the main rotating shaft. Therefore, analyzing vibrations and mitigating them in wind turbines is important. Nevertheless, traditional research on wind turbines has predominantly focused on enhancing aerodynamic performance, with limited attention given to the blade vibration analysis. Recently, studies have begun to explore the elastic structure of large wind turbine blades. Consequently, the exploration of the vibration characteristics of turbine blades is becoming an increasingly critical area of study. Resonance in wind turbine blades occurs because the excitation frequency due to aerodynamic forces is close to the natural frequency of one of the blade modes, resulting in dangerously large amplitudes of vibration. Analyzing this phenomenon requires further investigation to study the complex structural dynamics of rotating blades, considering the bend-twist coupling.

### Dynamic models and methodologies for solving nonlinear equations

Deformed and undeformed configurations of a blade modeled as a cantilever beam are exhibited in Figure 3. Rotating beam-like structures are widely used in various engineering applications, including those for helicopters, wind turbines, and compressor blades (Yang et al., 2017; Yardimoglu and Yildirim, 2004). In a broad sense, structural vibration issues are predominantly associated with the system’s natural frequencies (Houbolt and Brooks, 1958; Rao and Carnegie, 1970). Thus, for engineering design objectives, a comprehensive understanding of their dynamic properties is crucial (Yang et al., 2018).

Three main theories, documented in the literature, explain the dynamics of beams, including the Euler-Bernoulli, Rayleigh, and Timoshenko theories. The origins of the Euler-Bernoulli theory date back to the 18th century. Rotary inertia and shear deformation effects are neglected in Euler-Bernoulli beam theory. This theory typically results in an overestimation of the natural frequencies of a vibrating beam. Rayleigh beam theory considers the influence of rotational inertia associated with the cross-sectional area. While this offers some improvements to the Euler-Bernoulli beam model, it remains evident that the natural frequencies continue to be overestimated. The Timoshenko Beam Model differs from the Euler-Bernoulli model by adding the effects of shear distortion and rotational inertia. Table 1 offers an overview of the models.

**Table 1.** Comparison of three different beam models.

Beam theory	Lateral displacement	Bending moment	Rotary inertia	Shear deformation
Euler-Bernoulli	Yes	Yes	No	No
Rayleigh	Yes	Yes	Yes	No
Timoshenko	Yes	Yes	Yes	Yes

**Figure 8.**
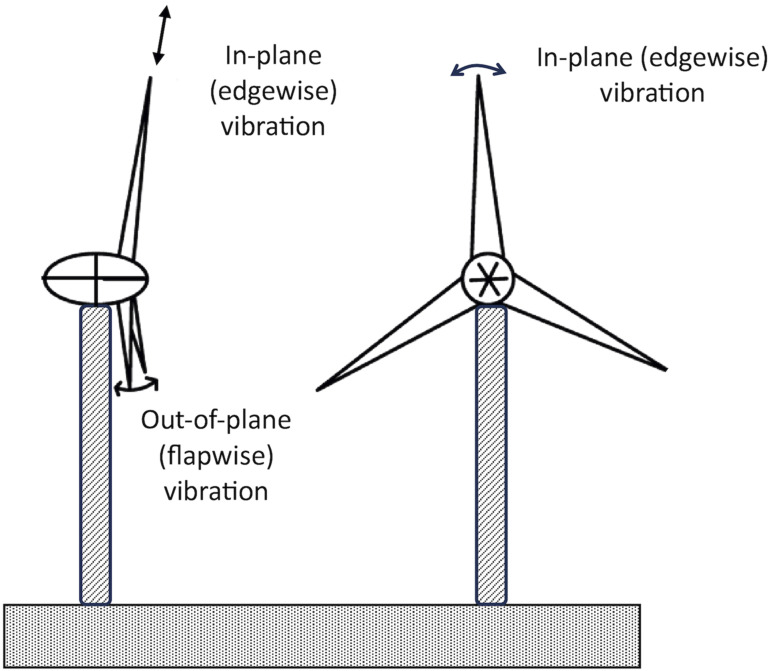
Depiction of edgewise and flapwise motions of a wind turbine blade.

The deformation of a wind turbine blade is generally studied in the axial, torsional, edge, and flap directions (Saram and Yang, 2025). When the pre-twist and the torsional motions of the blade are disregarded, the edgewise and flapwise deflections are called in-plane and out-of-plane deflections, respectively, as depicted in Figure 8. Jokar et al. (2021) developed a new dynamic model for the vibration analysis of HAWT blades that undergo both parametric and direct excitations of a harmonic nature due to gravitational and aerodynamic forces. A technique based on the pseudo arc-length continuation method is employed to analyze the amplitude-frequency response in multi-degree-of-freedom (DOF) systems. Thomas et al. (2016) examined the primary resonance of a rotating cantilever beam, considering significant deformations. Three different models were tested; the first two are derived from the beam’s analytical models. These two models are discretized using a suitable modal basis and solved using a numerical path-following method. The third model employs a finite-element discretization and is integrated over time. However, the main drawback of this work is the neglect of coupling with nonlinear aerodynamics, a significant factor in aeroelastic instabilities such as flutter. Huang and Zhu (2014) employed the incremental harmonic balance (IHB) method to examine a rotating Euler-Bernoulli beam attached to a rigid hub and subjected to gravitational forces. The studies by Jokar et al. (2021) and Huang and Zhu (2014) are valuable research tools in technical literature due to their emphasis on the theoretical foundation rather than practical tools for industrial design.

However, there is a lack of literature on the coupled flapwise and edgewise modes of vibrations. Rao and Carnegie (1970) examined the coupled system; however, their analysis was limited to determining the natural frequencies of a straight cantilever beam with an asymmetric cross-section. Their study did not incorporate cubic nonlinearities or explore the presence of various resonances. Likewise, Houbolt and Brooks (1958) have formulated the coupled system exclusively. The final equations of motion contain the unsteady aerodynamic forcing term and the cubic nonlinearities. In most cases, finding the closed-form solutions of the coupled differential equations is impossible. Most scholars have examined the two scenarios independently, rather than the coupled system, as Tang et al. (2015) have only explored the flapwise bending vibrations.

**Figure 9.**
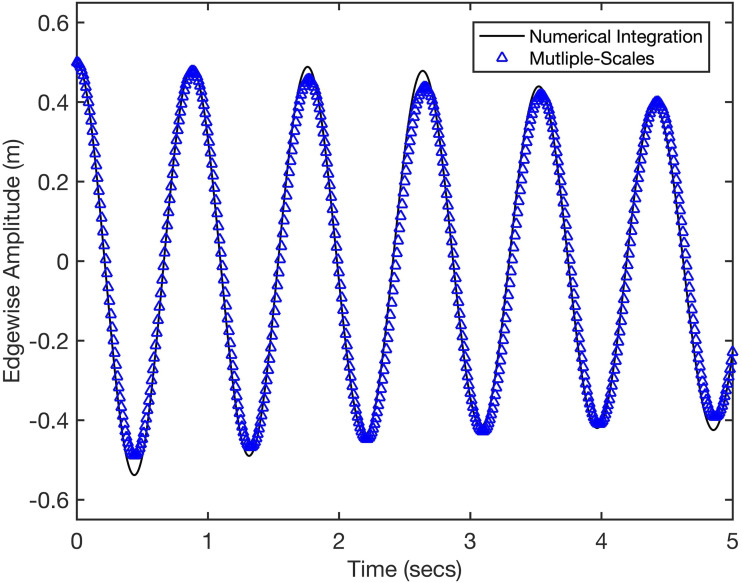
Comparison of numerical and analytical results for the non-resonant edgewise amplitude of a national renewable energy laboratory (NREL) 5-MW wind turbine blade.

Since many scholars have utilized the Euler-Bernoulli beam theory due to its simplicity and usefulness as a preliminary design tool, we modeled 5-MW HAWT reference blade from the National Renewable Energy Laboratory (NREL) as a cantilever beam using the Euler-Bernoulli beam theory to demonstrate its effectiveness. The first torsion mode, the first edge mode, and the first two flap modes are considered in this example for a reliable analysis. Final coupled model differential equations for the blade motions in flapwise and edgewise directions are given by:

(4) $${\ddot{\;q}}_{j}={b_{j,1}{\dot{q}}_{1}+b_{j,2}{\dot{q}}_{2}+b}_{j,3}q_{1}+b_{j,4}q_{2}+b_{j,5}q_{1}^{2}q_{2}+b_{j,6}q_{1}q_{2}^{2}+{\;b}_{j,7}q_{1}p_{1}^{2}+b_{j,8}q_{2}p_{1}^{2}+b_{j,9}q_{1}^{3}+b_{j,10}q_{2}^{3}+b_{j,11}\;\cos\left(\Omega t\right),j=1,2$$

(5) $${\ddot{p}}_{1}={f_{1}{\dot{p}}_{1}+f}_{3}p_{1}+f_{4}p_{1}q_{1}q_{2}+f_{5}p_{1}q_{1}^{2}+f_{6}p_{1}q_{2}^{2}+f_{7}p_{1}^{3}+f_{8}\;\cos\left(\Omega t\right)+f_{9}\;\sin\left(\Omega t\right)$$

where $b_{j,i}\;\text{and}\;f_{k}$ are the coefficients of the equations (4) and (5) and are defined in Table 2. Ω is the rotor speed, $q_{j}\;\text{and}\;p_{1}$ are deformations in flap and edge directions, respectively. Specifications of the 5-MW NREL wind turbine blade are adopted from Jonkman et al. (2009). The gross characteristics of this wind turbine are shown in Table 3. Figure 9 displays the time history graph of the first edgewise amplitude for both analytical and numerical results, which show good agreement, demonstrating the usefulness and effectiveness of the Euler-Bernoulli model.

**Table 2.** Coefficients $b_{j,i}$ and $f_{k}$ of equations (4) and (5) computed using the structural specifications of a reference National Renewable Energy Laboratory (NREL) 5-MW wind turbine.

Coefficients of equation (4)	Values	Coefficients of equation (4)	Values	Coefficients of equation (5)	Values
$b_{1,1}$	−0.05	$b_{2,1}$	−0.05	$f_{1}$	−0.05
$b_{1,2}$	−0.05	$b_{2,2}$	−0.05	$f_{3}$	−71.38
$b_{1,3}$	0.2306	$b_{2,3}$	123.3	$f_{4}$	−8.543
$b_{1,4}$	−325.0	$b_{2,4}$	−1024.1	$f_{5}$	−2.075
$b_{1,5}$	−27.08	$b_{2,5}$	−28.02	$f_{6}$	−17.71
$b_{1,6}$	−86.11	$b_{2,6}$	−64.98	$f_{7}$	−2.075
$b_{1,7}$	−3.294	$b_{2,7}$	−2.391	$f_{8}$	0.8
$b_{1,8}$	−9.027	$b_{2,8}$	−9.342	$f_{9}$	0.8
$b_{1,9}$	−3.294	$b_{2,9}$	−2.392	-	-
$b_{1,10}$	−95.32	$b_{2,10}$	−96.93	-	-
$b_{1,11}$	22	$b_{2,11}$	22	-	-

**Table 3.** General characteristics of a national renewable energy laboratory (NREL) 5-MW wind turbine.

General characteristics	Values
Rotor diameter (m)	123
Wind speed (m/s)	11.2
Rotational speed (rpm)	12.1
Slope of section lift curve	2 π
Cut-in and cut-out wind speed (m/s)	3, 25
Setting angle ( $\theta_{0}$ ), conning angle ( $\beta_{p}$ ) (deg)	2, −2.5
Blade length ( R )*,* hub height ( $h_{0}$ ) (m)	61.5, 90
x-component of distance from blade root to hub center ( $e_{x}$ ) (m)	1.5
y-component of distance from blade root to hub center ( $e_{y}$ ) (m)	0
Number of blades ( N ), drag coefficient ( $C_{D}$ )	3, 0.016

Table 4 presents the natural frequencies in the flap and edge directions obtained by previous scholars, who applied different beam theories to the NREL 5-MW wind turbine blade. The natural frequencies for the first flap and edge modes computed by various beam models closely resemble each other. However, compared to the geometrically exact beam model, the Timoshenko model predicted more accurate modal natural frequencies for flapwise modes than the Euler-Bernoulli model, especially in higher modes (the second flapwise mode). Second flapwise frequency computed by Yuan and Wang (2021) and Jonkman et al. (2009) using Euler-Bernoulli model differ by about 5.23%, Jeong et al. (2014) using Timoshenko model by 3.13%, and Algolfalt et al. (2022a) through Rayleigh model by 3.4% from the findings of Li et al. (2020) who employed geometrically exact formulations.

**Table 4.** Modal natural frequencies of national renewable energy laboratory (NREL) 5-MW computed using various beam theories.

Beam models	First flap (Hz)	First edge (Hz)	Second flap (Hz)	Citations
Euler-Bernoulli	0.67	-	1.96	Zhuang and Yuan (2024)
	0.718	1.1218	2.0148	Yuan and Wang (2021)
	0.6664	1.09	-	Acar and Feeny (2017)
	0.67	1.09	1.95	Tang et al. (2025)
	0.6993	1.0898	2.0205	Jonkman et al. (2009)
	0.69		1.99	Shakya et al. (2019)
Timoshenko	0.68	1.10	1.98	Jeong et al. (2014)
	0.67	1.09	1.95	Algolfat et al. (2022a)
	0.6704	1.0958	1.8992	Stanoev and Chandrashekhara (2019)
Rayleigh	0.68	1.11	1.98	Algolfat et al. (2022a)
	0.6808	1.1129	1.9851	Algolfat et al. (2022b)
	0.727	-	-	Jokar et al. (2020)
Geometrically exact beam model	0.67	1.11	1.92	Li et al. (2020)
	0.6830	-	1.9104	Rezaei et al. (2018)
	0.69	1.12	2	Jiang et al. (2022)

Since the Euler-Bernoulli model has negligible shear deformation and rotary inertia, it makes it simpler but less accurate for higher modes. On the other hand, the Timoshenko model considers shear deformation and rotary inertia, providing better accuracy for natural frequencies of higher modes and thick sections. A geometrically exact beam model can address significant deformation effects that beam models, such as the Euler-Bernoulli and Timoshenko models, cannot fully capture. Exact beam formulations provided accurate modal natural frequencies; however, they are computationally expensive and often limit their use to final design stages. The Euler-Bernoulli beam model remains a valuable tool for initial dynamic analysis of the blade due to its simplicity and computing efficiency. However, more comprehensive formulations, such as the Timoshenko model and exact geometric beam formulations, are essential for accurately predicting the natural frequencies of higher modes (second flapwise modes) and instability phenomena, including flutter and resonance.

The dynamical beam models discussed in this section provide the theoretical framework; however, their actual value is demonstrated when applied to the occurrence of resonance in wind turbines.

### Occurrence of resonance due to nonlinearities

Nonlinear effects could result in behaviors that reduce the efficiency of the wind turbine system and increase system instability. These phenomena comprise primary resonance, superharmonic and subharmonic resonances, bifurcation, and jump phenomena (Karimi and Moradi, 2018). A multitude of studies have been undertaken concerning the resonance of wind turbine blades, including parametric resonance (Chopra and Dugundji, 1979), internal resonance (Gilliatt et al., 2003; Larsen et al., 2007; Larsen and Nielsen, 2006, 2007; Li et al., 2014), sub and superharmonic resonances (Inoue et al., 2012; Li et al., 2012; Ramakrishnan and Feeny, 2012). Vibration of a system cannot be eliminated; however, it can be turned into energy or suppressed. Primary and internal resonances for flapwise vibration, considering geometric nonlinearity, were analyzed by Li et al. (2014) in response to an unsteady aerodynamic force. Ramakrishnan and Feeny (2012) developed partial differential equations for the in-plane vibration of a wind turbine blade subjected to cyclic gravitational forces using the extended Hamilton principle. They have included the nonlinear curvature and shortcoming terms and discussed the effects of several parameters on frequency response curves under super- and subharmonic resonances. Inoue et al. (2012) investigated the flapwise vibration characteristics of an elastic wind turbine blade subjected to wind and gravitational forces. A superharmonic resonance occurred due to the cubic nonlinear terms in the equation, and this was also experimentally validated. Talwekar et al. (2020) studied the steady-state response of an HAWT blade in the flap and edge directions under the influence of gravity and aerodynamic forces, considering the blade as an isolated elastic body, and did not consider the coupling between the modes. Superharmonic resonances were observed due to various nonlinearities in the system. Acar et al. (2016) identified the superharmonic and subharmonic resonances of orders 2 and ½ for in-plane vibrations influenced by gravitational forces. The relationships between amplitude and frequency, as well as the stability of these resonances, were examined. Zhou et al. (2021) investigated the primary resonance of a tapered, rotating beam subjected to a uniformly distributed load. Nonlinear partial differential equations are formulated by applying Hamilton’s principle. However, the analysis of Talwekar et al. (2020) has not considered the anisotropic material behavior and unsteady aerodynamic forces, and Zhou et al. (2021) have neglected the coupling with unsteady aerodynamic forces and damping that govern real-world blade behavior.

**Figure 10.**
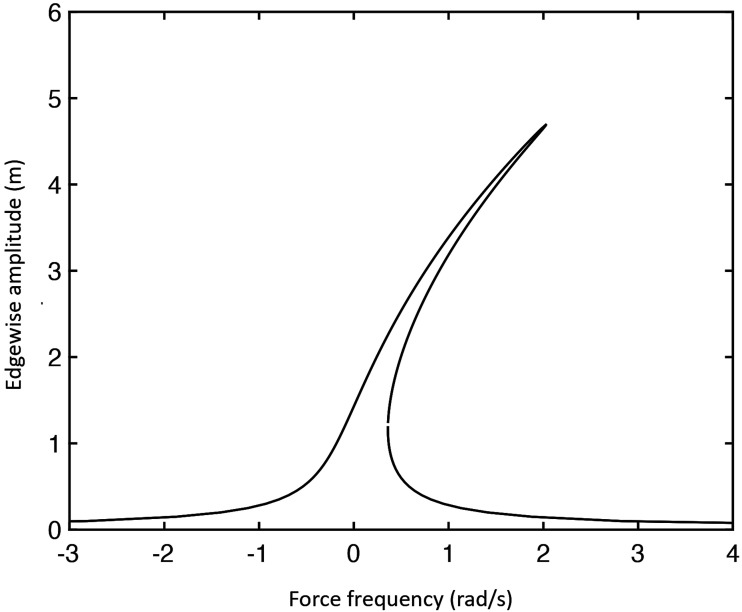
Nonlinear frequency response curve for the first edgewise amplitude under primary resonance (when the excitation frequency aligns with the blade modal natural frequency).

Primary resonance occurs when the excitation frequency aligns with the blade modal natural frequency. The first flap and first edge modes natural frequencies for a NREL 5-MW wind turbine blade are 0.6993 Hz and 1.0898 Hz, respectively (Jonkman et al., 2009). Therefore, wind turbine blades are likely to encounter primary resonance in real-world scenarios. The occurrence of primary resonance in the first edgewise amplitude for the NREL blade is depicted in Figure 10 through a frequency response curve, illustrating the hardening spring behavior resulting from the cubic nonlinearities.

Despite the works on resonance, none of the studies have included the combination resonance that occurs when internal and primary resonances overlap. Through the energy transfer, combination resonance can efficiently moderate the primary resonance. Beyond the excitation mechanism, the vibrational behavior of modern ultra-large and flexible blades gets even more complicated due to mode couplings, such as bend-twist coupling.

### Dynamics of blade vibrations considering bending-torsion coupling

The wind turbine blades’ edgewise, flapwise, and torsion vibration modes are coupled due to their aerofoil cross-section. Therefore, studying the effects of geometric and structural parameters on the coupled vibration modes is required. In a series of papers, the Hamilton principle was employed to develop nonlinear equations of motion for both extensional and inextensional beams, considering bending and torsion. It was observed that the nonlinear curvature terms play a significant role in the system dynamics, in addition to the inertia terms (Silva and Glynn, 1978). Li et al. (2016) have considered the coupling between axial, edgewise, flapwise, and torsion vibration modes of a wind turbine blade under the aerodynamic loading. Dokumaci (1987) developed a closed-form solution for the coupled bending-torsion vibrations of an Euler-Bernoulli beam and demonstrated how the slenderness and torsion-to-bending ratios affect the natural frequencies. Bercin and Tanaka (1997) modeled the Timoshenko beam for coupled bending-torsion vibrations, including shear deformations, rotary inertia, and warping effects in the coupled equations of motion. Bishop and Price (1977) investigated the coupled bending-torsion vibrations, considering the effects of rotary inertia and shear deformation. Subrahmanyam et al. (1981) modeled the blade as a Timoshenko beam and examined the coupling of bending and torsion in rotating blades with asymmetric cross-sections. The use of a simple uniform beam with symmetrical cross-section and linear elastic material hindered the applicability of the works of Dokumaci (1987), Bercin and Tanaka (1997), Bishop and Price (1977), and Subrahmanyam et al. (1981) to modern non-uniform blades, where the mode coupling and pre-twist are useful design features.

Ozgumus and Kaya (2013) conducted free vibration analysis of a rotating, double-tapered Timoshenko beam with coupled flapwise-torsion vibrations. They included the parameters of hub radius, rotor speed and inertia, shear deformation, slenderness, and taper ratios. The influence of these parameters on the natural frequencies was examined. Sinha (2006) computed the natural frequencies for beams with varying twist angles, aspect ratios, and slenderness ratios, considering the bending-torsion-axial coupling. Banerjee and Rao (1976) presented both analytical and experimental analyses to calculate the natural frequencies of a blade mounted on a rotating disk with coupled bending-torsion motions. The equations of motion mentioned in most previous research are partial differential equations, and closed-form solutions are usually not attainable. Numerous numerical techniques have been developed for solving these partial differential equations. Although numerical studies incorporating the torsional degree of freedom thoroughly investigate the dynamic behavior of blades, they often demand significant computational costs.

Most of the work in technical literature has overlooked the torsion mode when studying coupled-mode systems. Research on the vibration characteristics of a three-mode coupled system, comprising flap, edge, and torsion modes, is limited. Silva and Glynn (1978) have developed equations that consider only two bending modes and torsion, without including cubic nonlinearities in the differential equations. Therefore, there is a need to analyze comprehensive dynamic modeling that incorporates torsional vibration in the system. An internal resonance condition that involves the first two flap modes, the first edge mode, and the first torsion mode can be addressed. Furthermore, a combination resonance scenario, in which both internal and primary resonances occur, may be investigated.

The modeling of primary and internal resonance occurrence provides the basis for understanding wind turbine blade vibrations. When aerodynamic forces cause the blade to undergo self-excited oscillations instead of responding to external forces, the blade enters aeroelastic instabilities, presenting its unique challenges. Stall-induced vibration (SIV) and classical flutter are investigated in more detail in the next section, highlighting the possibility of these instabilities occurring on wind turbines.

## Aeroelastic instabilities

Aeroelastic instability is a self-excited vibration resulting from the interactions between aerodynamics, dynamics, and elasticity (Men et al., 2024). Classical flutter and Stall-induced vibrations (SIV) are the two primary forms of aeroelastic instability observed in wind turbines. Men et al. (2024) pointed out that, to date, during the parking and installation stages of wind turbines exceeding 10 MW, the phenomenon of aeroelastic instability has been observed. They further illustrated that the aerodynamic loadings have been overlooked in conventional research. Aeroelastic instability is challenging to track and quantify.

### Classical flutter

**Figure 11.**
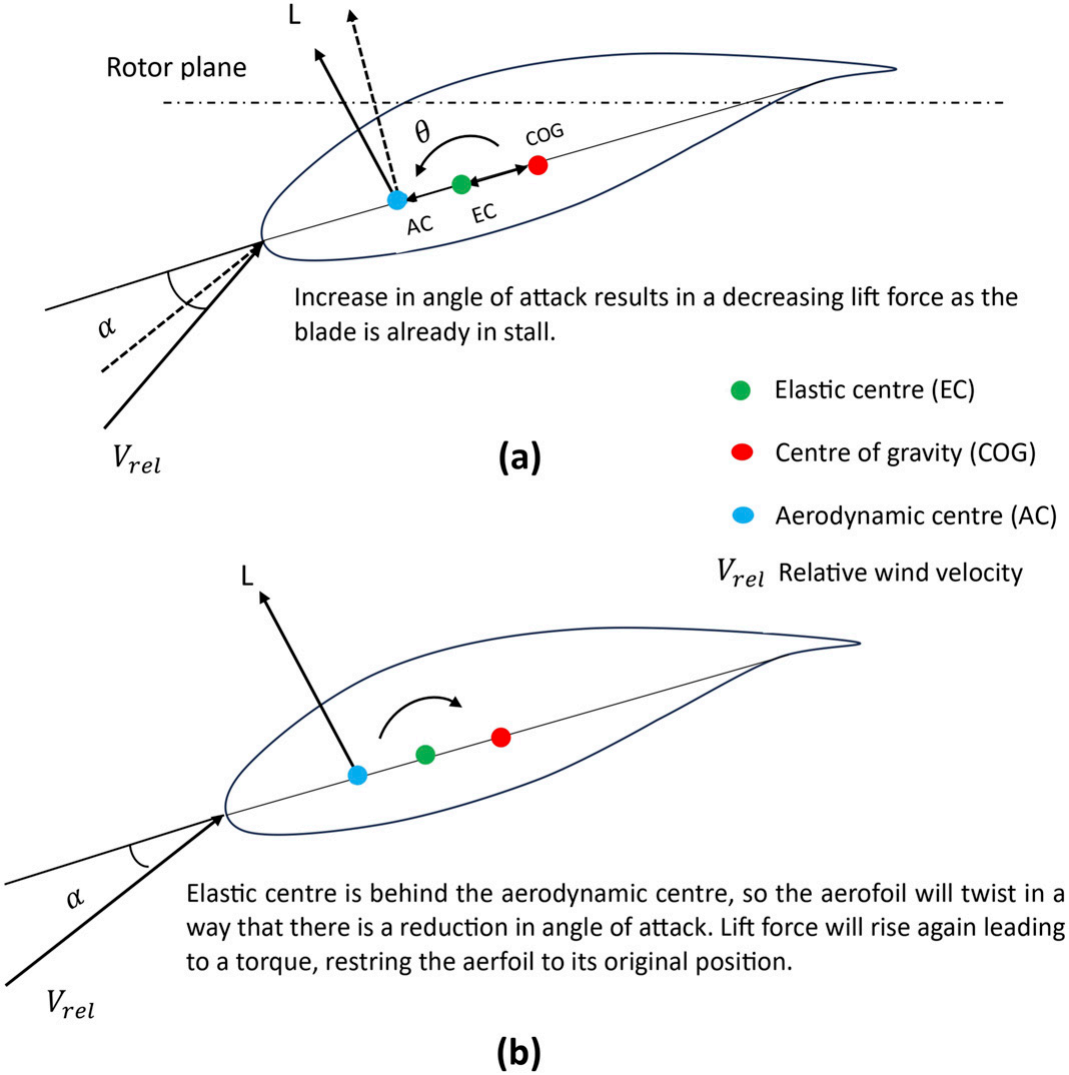
Mechanism of stall flutter instability on an aerofoil: showing the corresponding changes in angle of attack ( α ), lift force ( L ), and aerofoil twist ( θ ).

The slender design of existing wind turbine blades increases their sensitivity to aeroelastic instabilities. As a result, aeroelasticity-based criteria are becoming increasingly significant in the design of these blades. Stability is more likely to be prioritized in wind turbine design than load considerations (Bir and Jonkman, 2007). Classical flutter instability is a self-excited phenomenon that happens when torsional oscillations are coupled destructively with flapping oscillations, resulting in divergent oscillations that may lead to potential structural failure. Stall flutter on an aerofoil is demonstrated in Figure 11. Due to their considerable torsional stiffness, wind turbines are unlikely to experience this instability under typical operating conditions. However, if a flexible pitch control system or a pitch connection malfunction is present, it must be considered (Eggleston and Stoddard, 1987). The classical flutter may arise when the flow remains attached, the tip speed is high, the torsional stiffness is relatively low, and the center of gravity is behind the aerodynamic center (Hansen, 2007).

Even though it has not been reported on the commercial wind turbines yet, it is still possible that larger blade designs will be used in the future, particularly for offshore wind turbines that may operate at high tip speed ratios (Hansen, 2007). Therefore, designing an aeroelastic model capable of accurately forecasting aeroelastic instabilities is important. These models are vital not only for stability analysis but also for facilitating effective blade design and operational control, thereby improving the aeroelastic properties of the blades. Many researchers have explored the flutter instability of an isolated blade. Flutter speed of a 20-kW HAWT rotor with a stiff blade measuring 5 m in radius is considerably higher than its operating speed, as demonstrated by Lobitz and Veers (1998). However, for a 1.5 MW blade having a radius of 35 m, the flutter speed is almost double its operating speed (Lobitz, 2004). The gap between flutter speed and operating speed diminishes as the blade size grows. This observation has led to numerous studies into the flutter behavior of larger wind turbine blades. Lobitz (2005) examined the parameter sensitivities influencing the flutter margin of the WindPACT blade. The results suggested that changes in torsional rigidity and the position of the center of gravity with respect to the shear center axis have an impact on the flutter speed. The analysis by Lobitz (2005) is based on linear models, overlooking the crucial nonlinear effects due to large deformations and structural damping that are typical of real-world turbine operations. Additionally, while correctly identifying the problem for future large-scale blades, the specific quantitative results, such as the flutter speed being twice the operating speed for a 35 m blade, were relevant to the technology of that era. Owens et al. (2013) developed a specialized tool for exploring the aeroelastic stability of blades using the finite-element method. His formulation encompasses both centrifugal and gyroscopic effects, as well as the coupling resulting from composite material configurations. Theodorsen’s unsteady model was applied to derive the aerodynamic loading. The findings revealed a considerably greater flutter margin for the large blade compared to the forecasts made by Lobitz (2004). The works of Owens et al. (2013) were necessarily based on design predictions, as blades were still conceptual. As a result, the study could not be verified against full-scale wind turbine experimental results; the issue of the most accurate modeling technique remained unanswered.

Zhou et al. (2018) analyzed the flutter performance of a wind turbine blade exhibiting coupled bend-twist motions by integrating the Blade Element Momentum (BEM) theory and an unsteady dynamic stall model with a nonlinear beam model. The study found that an imbalance in the skin laminates did not significantly affect the flutter performance. A critical limitation of their work is that it relies on simulated models without experimental validation. This leaves the question of how these findings affect other load cases and the overall fatigue life. Hayat et al. (2016) conducted a transient analysis to estimate flutter, including the bend-twist stiffness coupling of the composite beam in their analysis. The results revealed that incorporating carbon fiber into the composite blade improves flapwise and torsional stiffness while reducing the flutter instability. This study also did not quantitatively compare the flutter margin gained through bend-twist coupling against the other established methods, leaving the uncertainty of its relative cost-benefit advantage.

The aerodynamic model used has an impact on the predictability of flutter speed. Steady aerodynamic models tend to exhibit lower flutter speeds compared to unsteady models (Hafeez and El-Badawy, 2018). For high rotor speeds, the outcomes of Theodorsen’s theory differ significantly from those of time-domain models, such as the BEM model, vortex models, and CFD models (Hayat and Ha, 2015). Pourazarm et al. (2016), by employing the Theodorsen’s model, noted that a rise in the flapwise frequency can lead to a reduction in flutter frequency.

**Figure 12.**
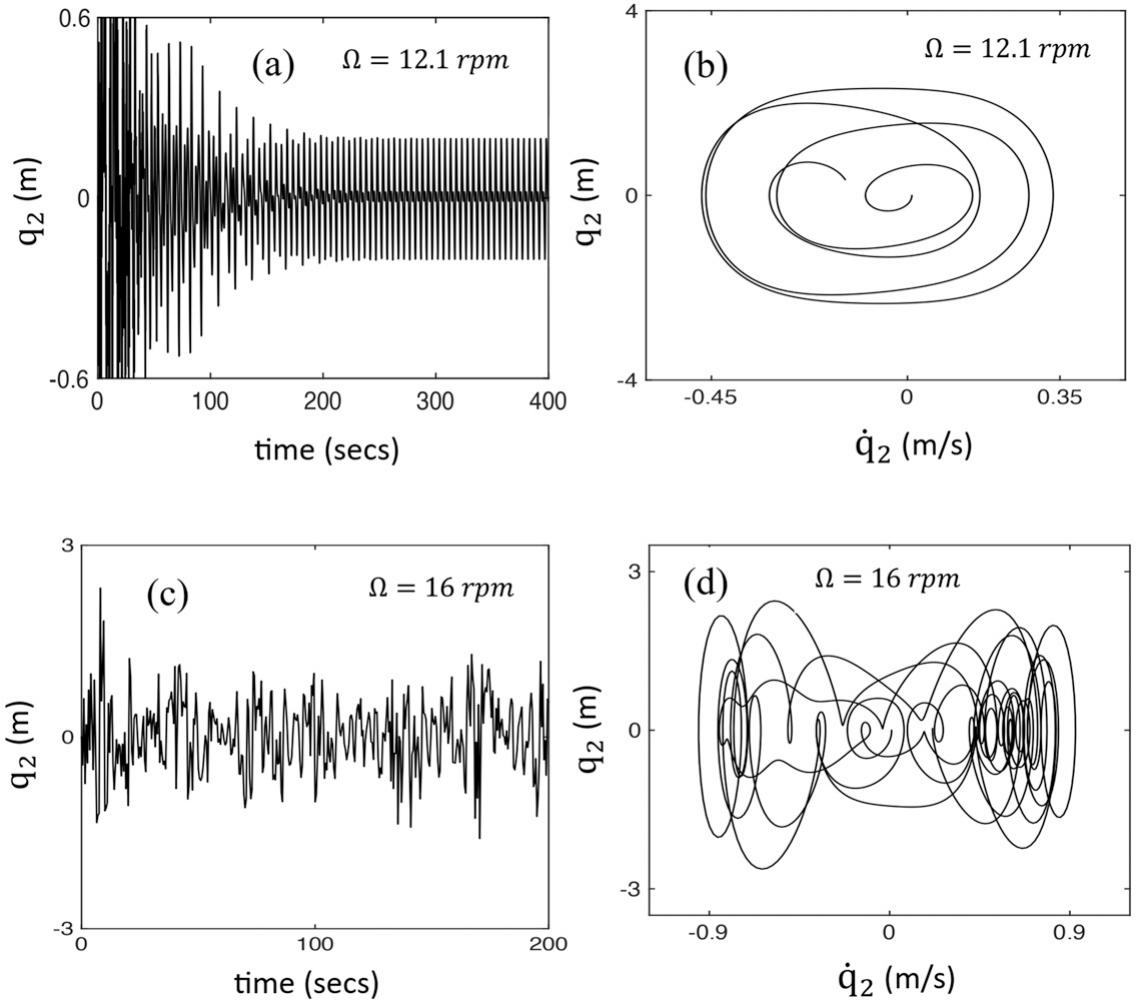
Time history and phase graphs for the second flapwsie amplitude of a National Renewable Energy Laboratory (NREL) 5-MW wind turbine blade for two different rotor speeds: (a) time history graph at rotor speed Ω=12.1 rpm ; (b) phase plot at rotor speed Ω=12.1 rpm ; (c) time history graph at rotor speed Ω=16 rpm ; (d) phase plot at rotor speed Ω=16 rpm .

Flutter speed is a crucial parameter when analyzing the aeroelastic instabilities of wind turbine blades, as highlighted by the works of Lobitz and Veers (1998), Lobitz (2004), and Lobitz (2005). The flutter speed of an NREL 5-MW blade with coupled modes of vibration, as calculated using the Euler-Bernoulli model, is presented as an example in this work. Figure 12 displayed the phase diagrams and time history response for first flapwise (out-of-plane) mode of NREL 5-MW reference wind turbine blade at rotor speeds of Ω=12.1 rpm and Ω=16 rpm . It was noted that at rotor rated speed Ω=12.1 rpm flapwise mode is stable, flutter instability occurred at rotor speed Ω=16 rpm .

Table 5 shows the flutter speed reported in the literature by various researchers for the NREL 5-MW and International Energy Agency (IEA) 15-MW reference wind turbines. Hansen (2004) and Shakya et al. (2019) predicted the flutter speeds for NREL 5-MW using Euler-Bernoulli models, which overestimated the flutter speeds by 38% compared to the findings of Meng (2011), whose geometrically exact beam model incorporated shear deformation and nonlinearities. Similarly, Qian et al. (2024) computed the flutter speeds as 17.44 rpm for the IEA 15-MW ultra-large wind turbine using the Euler-Bernoulli model, which overestimates the result compared to the findings of Li et al. (2023), who used geometrically exact formulations, as 12.88 rpm. This is because Euler-Bernoulli models fail to capture bend-twist coupling, which is essential for accurately capturing flutter instability. Furthermore, models incorporating shear deformation (Timoshenko) and nonlinearities (geometrically exact formulations) could accurately capture the negative damping that causes flutter. Therefore, designers should adopt geometrically exact formulations for final design validation, especially for modern flexible blades that undergo large deformations or operate near resonance.

**Table 5.** Flutter speed of national renewable energy laboratory (NREL) 5-MW and international energy agency (IEA) 15-MW wind turbines computed using various beam models.

Wind turbine models	Beam models	Flutter speed (rpm)	Citations
NREL 5-MW	Euler-Bernoulli	26.4	Shakya et al. (2019)
		20.7	Pourazarm et al. (2016)
	FEM-based approach	24	Hansen (2004)
	Timoshenko	22.3	Hayat and Ha (2015)
	Geometrically exact formulations	19.1	Meng (2011)
IEA 15-MW	Euler-Bernoulli	17.44	Qian et al. (2024)
	Geometrically exact formulations	12.88	Li et al. (2023)

The majority of the aeroelastic instability studies documented in the literature have predominantly used incompressible flows for flutter investigations of blades. Nevertheless, during flutter occurrence for longer blades, the relative velocities at sections away from the hub often fall within the subsonic compressible region (Mach numbers surpassing 0.3). Therefore, to accurately account for the compressibility effects, CFD-based methods may be employed. However, these methods tend to be computationally high-priced and are not ideal for addressing frequency-domain solutions related to aeroelastic instabilities. The unsteady BEM method, which requires significantly less computational power compared to CFD models, allows for the incorporation of dynamic wake and stall models, making it an even more efficient option and applicable to one-dimensional beam models. Limited studies have examined the impact of shifting the centroid towards the leading or trailing edge on reducing flutter limits. The models should incorporate the mass, shear, and aerodynamic centers’ chordwise offsets.

In contrast to the negative damping of SIV, the classical flutter instability is a more fundamental coupling between structural modes resulting from unsteady aerodynamics. The classical flutter is a well-known issue for aircraft wings; paying attention to its occurrence in wind turbines is becoming increasingly important. Stall-induced vibration (SIV) at lower wind speeds is another critical issue this shift highlights.

### Stall-induced vibrations (SIVs)

**Figure 13.**
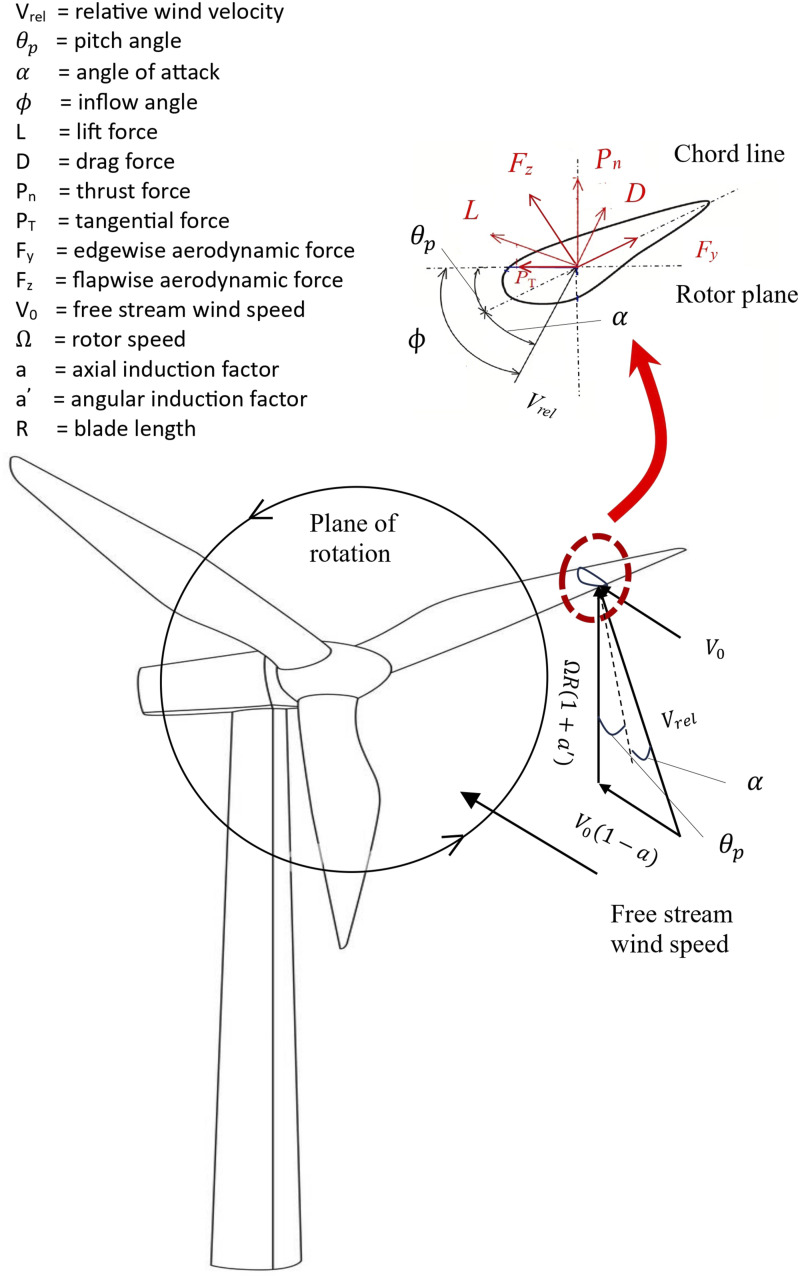
Three-blade wind turbine system and sectional aerodynamics of a single blade: decomposition of velocities and aerodynamic forces on an aerofoil cross-section.

SIV represents an aeroelastic instability that may occur when large sections of the blade experience moderate stall (further increase in angle of attack results in decreasing lift force), resulting in substantial internal loads. The negative lift observed (negative slope of the section lift curve) in this area results in a negative aerodynamic damping in the blade sections, causing the lift force to be in phase with the velocity of the oscillating blade (Dowell, 2015). A model is presented in Figure 13 to determine the aerodynamic damping of an aerofoil, which illustrates the velocities and forces acting on the aerofoil’s cross-section. Aerodynamic damping in edgewise and flapwise directions is given by Rasmussen et al. (1999) as:

(6) $$c_{yy}\left(r\right)=\frac{\rho_{a}c_{l}r\Omega }{2V_{rel}}\left[r\Omega \left(2+\frac{{V_{0}}^{2}}{r^{2}\Omega^{2}}\right)C_{D}-V_{0}\frac{dC_{d}}{d\alpha }-V_{0}C_{L}+\frac{{V_{0}}^{2}}{r\Omega }\frac{dC_{L}}{d\alpha }\right]$$

(7) $$c_{zz}\left(r\right)=\frac{\rho_{a}c_{l}r\Omega }{2V_{rel}}\left[r\Omega \left(1+\frac{2{V_{0}}^{2}}{r^{2}\Omega^{2}}\right)C_{D}+V_{0}\frac{dC_{d}}{d\alpha }+V_{0}C_{L}+r\Omega \frac{dC_{L}}{d\alpha }\right]$$

where $c_{yy}$ and $c_{zz}$ are the aerodynamic damping in the edgewise and flapwise motions, respectively. $c_{l}$ is the chord length, r is the radial distance of the blade section from the hub, $\rho_{a}$ is the air density, $V_{rel}$ is the relative wind velocity, Ω is the rotor speed, $V_{0}$ the free stream wind velocity, α is the angle of attack, $C_{L}$ is the lift coefficient, and $C_{D}$ is the drag coefficient.

Equation (7) indicates that the flapwise motion is typically well-damped; however, there is a potential for reduced damping during stall, as the rate of change of $C_{L}$ with respect to α turns negative beyond the stall point. Should this damping become negative, it would cause SIV, which could reduce the blade life. Equation (6) indicates that negative damping of the edgewise motion can occur, another SIV, which has been identified as a problem for turbine blades (Stiesdal, 1994). Therefore, the first edgewise mode must not represent a purely in-plane mode. It must incorporate sufficient flapwise motion to ensure that the positive damping from this mode is adequate for achieving positive damping in edgewise mode. However, this does result in a decrease in the first flapwise mode damping. Over the last decade, edgewise SIVs have occasionally been noted in three-bladed wind turbines (Hansen, 2003). In addition to the potential danger for these vibrations to intensify, they also play a critical role in the fatigue loading of the blades, resulting in a decreased lifespan.

Researchers studied the stability of wind turbine blades considering the effects of aerodynamic damping. A tool for aeroelastic calculations was developed by Hansen (2004) to execute stability analysis of a wind turbine under different operating conditions. A close agreement was found between the measured and calculated outcomes of aeroelastic damping of a stall-regulated wind turbine. Although the study is an excellent tool for preliminary design due to its linear eigenvalue analysis, high-fidelity CFD nonlinear simulations are often necessary to ensure forecast accuracy for wind turbines operating in real-world conditions. In another study, Chen et al. (2017) developed a wavelet-based linearization method for calculating the aerodynamic damping of a wind turbine in operation. Hulskamp et al. (2011) developed a smart rotor featuring trailing-edge flaps aimed at reducing the load fluctuations and increasing the damping of the blade modes. This resulted in a notable decrease in dynamic loads. The complex integration of actuators and sensors for trailing-edge flaps introduces uncertainty into the fundamental aeroelastic phenomena. Skrzypiński and Gaunaa (2015) investigated the SIV that may occur in standstill wind turbine blades. Their findings revealed that the aerodynamic characteristics of their profiles had a significant influence on the stability of the blades. Since turbulent flow and different angles of attack along the blade span are the leading causes of instability in parked-state wind turbines, the simplified setup by the authors ignores these parameters in favor of an idealized uniform inflow. The work is better viewed as a first step towards improving high-fidelity aerodynamic models, rather than serving as a direct guide for blade designers, due to its highly simplified 2D aerofoil design approach. Damping characteristics and resonant frequencies of a parked offshore wind turbine were measured experimentally by Devriendt et al. (2014). A study conducted by Hansen (2003) involving a 600-kW turbine has demonstrated that the backward whirling mode of the edgewise vibrations exhibit lower aerodynamic damping compared to the forward whirling mode. According to his study, the observed variation in aerodynamic damping can be explained by the differing vibrations of the blades for the forward edgewise whirling mode and the backward edgewise whirling mode. He concluded that altering the mode shapes of wind turbines could prevent SIV. The use of linear aeroelastic models by Hansen (2003, 2004), and Hayat et al. (2016) may not be able to capture the complex nonlinear stall and damping effects near the actual flutter boundary.

Meng et al. (2025) simulated the stall-induced instability for an NREL 5-MW floating offshore wind turbine (FOWT) at standstill conditions. Using frequency-domain simulations, this instability was studied using linear and nonlinear aeroelastic models for various yaw and azimuth angles, which observed edgewise stall-induced instability due to negative aerodynamic damping at specific azimuth and yaw angles. They have also suggested some active control strategies to mitigate this instability type, demonstrating the effectiveness of the proposed methods through numerical simulations. The development of their model represents a significant contribution to the floating offshore wind turbines (FOWTs). However, focusing on a single platform type (spar-buoy) may limit its usage to other generalized FOWT platforms, such as semi-submersibles or tension-leg platforms.

**Figure 14.**
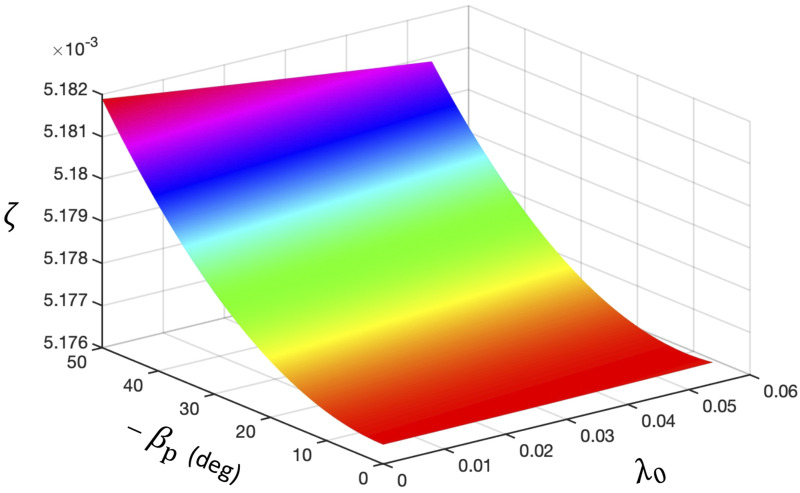
Plot of aerodynamic parameters for the parametric analyses of steady-state first edgewise amplitude during primary resonance: inflow ratio ( $\lambda_{0}$ ) and conning angle ( $\beta_{p}$ ) versus dimensionless damping ratio ( ζ ).

In previous works in this subsection, SIV was observed in wind turbine blades due to negative edgewise aerodynamic damping at specific inflow conditions and conning and yaw angles. Therefore, the influence of wind speed on the aerodynamic damping of the steady-state edgewise response of a reference NREL 5-MW wind turbine blade is studied as an example by using Greenberg’s formulations (Greenberg, 1947). A dimensionless ratio $\zeta =c_{yy}/2\sqrt{m_{yy}k_{yy}}$ is introduced for the damping of the *kth* mode. Damping ratio ζ dropped with increase in the inflow ratio $\lambda_{0}$ , and damping ratio decreased with increasing conning angle $\left|\beta_{p}\right|$ for a fixed $\lambda_{0}$ in case of first edgewise amplitude a possible indication of a stall-induced vibration (SIV), as illustrated in Figure 14.

While numerous studies have examined the structural dynamics and stability of wind turbine rotors, there are few studies related to the mitigation of stall-induced vibrations in wind turbine rotors during idling conditions, particularly through the theoretical and experimental approaches. The current models used in technical literature to predict the SIV in standstill wind turbine rotors have not taken into consideration the stochastic nature of aerodynamic forces. This oversight arises from the complex vortex structures generated by the blades when the airflow around the aerofoils becomes fully separated. Though the added damping effect provided by stochastic aerodynamic forces is negligible in practical applications, there may be other aerodynamic phenomena associated with deep-stall conditions that could affect the SIV in a standstill system. Furthermore, the modal dynamics of the complete turbine play a crucial role in stability evaluations; focusing solely on the analysis of blades may not be sufficient. Enhancing the modal dynamics may help prevent SIV.

Surrogate models have been employed in related applications that require expensive evaluations, including the assessment of site-specific loads (Dimitrov et al., 2018) and the investigation of the influence of various parameters on blade design (Barlas et al., 2021). The quantity of research examining the impact of inflow variables on SIV through surrogate models is quite limited. Santhanam et al. (2022) employed an Artificial Neural Network (ANN) model to efficiently investigate the dynamics of SIV within the inflow space, which includes five parameters related to wind. However, various machine learning algorithms, including fuzzy logic, K-Nearest Neighbors (KNNs), and Convolutional Neural Networks (CNNs), can be employed, along with additional parameters tailored to inflow conditions and aerofoil characteristics.

### Experimental versus computational studies on aeroelastic instabilities

Validating computational models through experimental methods is a key aspect of aeroelasticity research, as it provides confidence in making accurate predictions. Gao et al. (2025) compared the stall-induced vibrations reproduced through CFD-CSD simulation with wind tunnel measurements of the DU91-W2-250 aerofoil under active trailing edge windward conditions. They found good agreement in vibration characteristics, such as the growth of amplitude with wind speed and frequencies aligning with the blade’s natural modal frequencies. Subsequently, this hands-on experimental validation boosts confidence in developing an aeroelastic reduced-order model (ROM) by coupling structural dynamics and an unsteady aerodynamic model. However, the authors have exclusively focused on the two-dimensional aerofoil model, which limits the applicability of full 3D blade models. Real wind turbine blades undergo spanwise flow, rotational effects, and 3D structural dynamics that may change the instability characteristics observed in two-dimensional aerofoils.

Dai et al. (2023) utilized experimental data to evaluate the CFD-based predictions for stall flutter, focusing on the critical flutter speed and limit-cycle oscillation amplitudes. They developed a cutting-edge, multi-layer gated recurrent unit (GRU) neural network-based reduced-order model (ROM) to enhance calculations of aerodynamic forces. However, the fundamental limitations of machine learning algorithms are that they depend on the quality and quantity of the training data. The effectiveness of the GRU network developed by Dai et al. (2023) is limited by the range and diversity of conditions used in the CFD-generated training data.

Men et al. (2024) employ a different validation approach by comparing their results from OpenFAST with the published modal analysis of the DTU 10-MW reference wind turbine. Although this method provides some evidence of proper validation, it indirectly validates the model rather than directly confirming the predictions related to aeroelastic instability. Additionally, they examined how various parameters impact the aeroelastic instability of FOWT to determine its boundaries. This research used the BEM theory to model aerodynamic loads, which cannot account for the flow separation phenomena associated with dynamic stalls. Overlooking the dynamic stall effects could impact the stall-induced vibrations at high angles of attack.

Chen et al. (2022) performed experimental investigations using a NACA 0012 2D aerofoil model with pitching freedom to suppress stall flutter. They used time-resolved particle image velocimetry to capture the dynamic flow fields during stall-induced vibrations. The experimental method proved the effectiveness of flow control, which could help validate CFD models for active flow control strategies. Naung et al. (2025) introduced a nonlinear frequency-domain approach to examine the aeroelastic behavior of a complete wind turbine model. While showing excellent agreement with experimental data for aerodynamic damping and unsteady pressure distributions, the frequency-domain approach reduced the computational time to 9 hours compared to 120 hours for the time-domain method. Additionally, a complex, unsteady pressure distribution was observed at 324.5 r/min compared to 424.5 r/min, directly affecting the aerodynamic damping, and highlighting the importance of operational conditions on aeroelastic stability.

The hurdles to experimental validation in wind turbine aeroelasticity are substantial, especially for full-scale wind turbines, where controlled testing can be challenging due to the complex nature of the fluid-structure interactions involved. The validation challenges FOWT face are even more significant because of the added complexities of wave-structure interactions. Computational approaches offer the advantage of detailed parametric studies under carefully controlled conditions that would be difficult or impossible to achieve experimentally. Computational methods also benefit from conducting in-depth parametric studies in carefully controlled environments, which are challenging or impossible to achieve through experimental means. The computational cost of high-fidelity CFD methods is still significant, particularly when simulating complete wind turbines under turbulent inflow conditions.

## Vibration mitigation strategies

Structural control methods are typically employed in the vibration mitigation of innovative structures, such as bridges, buildings, and wind turbines. The vibration control of wind turbine blades involves modifying the blade’s shape, external structure, materials, or installation methods, as well as structural damping or attenuating aerodynamic loads, to reduce vibration and enhance the performance and service life of wind turbines. Three primary categories of control schemes include passive, active, and semi-active control systems.

### Passive control

The passive control scheme modifies the blade stiffness distribution, damping, and natural frequency to avoid coupling resonance with the system, or it can also redesign the blade structure and change the blade material directly to prevent flutter. Hayat and Ha (2015) attempted to enhance the aeroelastic stability of the NREL 5-MW wind turbine model by changing the ply angle of the composite material. They demonstrated that their model could improve the bending stiffness of the blades while reducing the torsional stiffness, hence lowering the critical flutter speed of the structure. Larwood et al. (2014) investigated the flutter behavior of an NREL 5-MW wind turbine with swept blades. The elastic axis of this blade type is curved compared to traditional blades, and the results indicate that it can contribute slightly to vibration suppression. However, this blade type improves flutter suppression capabilities while introducing significant fatigue problems. Hansen (2007) and Zhuang and Yuan (2024) introduced the mass redistribution of the blade by shifting the chordwise position of the center of gravity to influence flutter and reduce instability risks.

Sun et al. (2023) investigated the passive aeroelastic coupling of IEA 15-MW large and flexible wind turbine blades through bending and torsion coupling, as well as geometric sweep coupling, to reduce loads. The bend-twist coupling is achieved using off-axis composite fibers of the blade spar cap, while the geometric-sweep coupling is established by delineating the blade reference axis sweep curve. The findings show that these two couplings can reduce the load at the blade root by decreasing the off-axis angle of the optical fiber, thereby further decreasing the axial torque.

Liao et al. (2025) developed a bidirectional tuned mass damper (TMD) system for flutter suppression in the IEA 15-MW ultra-large offshore wind turbine blades, utilizing a finite-element model that accounts for geometric and material nonlinearities. Bidirectional TMD demonstrated significant effectiveness in reducing steady-state amplitude flutter compared to uncontrolled blades or unidirectional dampers. However, the additional mass on the blade and maintenance difficulties in harsh offshore conditions may pose significant challenges to the effective implementation of this scheme.

The finite-element method establishes a blade flutter characteristic equation based on the Euler-Bernoulli beam theory, combined with Theodorsen’s unsteady aerodynamic loads, as presented by Zhuang and Yuan (2024). Using NREL 5-MW wind turbine blades as an example, the influence of parameter changes in different regions of the blades on flutter characteristics was analyzed. Results indicated that parameter changes in the blade tip region have the most significant impact on flutter characteristics. However, the model employed an isotropic beam assumption, neglecting the anisotropic properties of composite materials commonly used in wind turbine blades, which may potentially oversimplify the blade’s structural dynamics. Furthermore, the model cannot capture the large deformations of ultra-long NREL 5-MW wind turbine blades because of the linear equations. Despite these limitations, the parametric framework provides valuable insights for initial blade design. However, it is necessary to use higher-fidelity models, such as CFD-FEM coupling or geometrically exact beam models, for a reliable assessment of aeroelastic stability.

Based on the CFD-FEM coupling methods, Yao et al. (2025) employ numerical simulations to investigate the aeroelastic response of the NREL 5-MW wind turbine blades when the blade pitch system fails in a typhoon environment. The blades were designed with composite layups, and the shutdown strategy for active typhoon resistance was proposed. The study found that adjusting the yaw and azimuth angles can significantly reduce the wind load on the blades, decrease blade tip vibrations, and improve stress concentration on the wind turbine blades. The study addresses a crucial practical problem of blade pitch system failure under extreme weather conditions, which is likely to occur for large offshore wind turbines in typhoon-prone regions. However, full blade stopping may not be feasible in all blade failures. Further, the progressive damage accumulation that might occur before the turbine reaches the recommended parking position has not been considered, potentially underestimating damage risks.

Passive control methods offer reliability and simplicity, as they require no external power or control mechanisms; however, they typically provide fixed performance characteristics that may not be ideal for all operating conditions.

### Active control

An active control features a damper and a real-time control system, which can provide real-time suppression of a broad excitation spectrum. The performance of this system also depends on how well the control algorithm works. Recently, active control systems and related control algorithms have garnered significant research interest due to technological advancements enabling faster real-time calculations. The promising active control strategies include active flow control, pitch control, and active damping control techniques.

Many academics have investigated the unloading techniques of wind turbine blades based on microtab structures. Macquart and Maheri (2015) investigated the impact of microtab methods on load reduction using intelligent control techniques and CFD analysis. The results show that the aerofoil lift calculated through the microtab is within the normal working range; however, the lift will be reduced beyond this range. Concurrently, with increased drag resistance, the circulation of the aerofoil changes. Therefore, deep-stall vibration may have an adverse effect. In addition, some scholars have combined microtabs with the trailing-edge flap (TEF) load reduction method to enhance the performance of blades, as they can generate greater lift and delay stall occurrence. Ye et al. (2021) tested this combined microtab and TEF technique for the NREL’s S809 blades. Due to the opposite direction of the vortices, the maximum lift coefficient increased by 25% and the airflow stall was delayed, significantly outperforming standard aerofoil testing.

Pitch control modulation involves adjusting the blade pitch angles to alter aerodynamic loading and mitigate the development of instabilities. A multivariable independent pitch control framework, which models the coupling between blades to provide frequency-domain response characteristics, was developed by Yuan et al. (2020). They applied a structural singular value synthesis approach to reduce periodic loads. However, as the blade size continues to increase, factors such as blade weight, reverse aerodynamic effects, and stall at extreme wind speeds have substantially raised the reliability challenges associated with pitch control techniques. Hence, it is not a standalone load-shedding strategy for large flexible blades under extreme wind loads.

Du et al. (2024) developed a control co-design optimization approach tailored explicitly for floating offshore wind turbines. Their approach simultaneously optimized the structural design and control parameters, explicitly considering aeroelastic-control couplings traditionally neglected. It was specifically the integrated optimization of the turbine’s fundamental active pitch and torque control systems within a co-design framework. The vibration and load reduction were achieved, resulting in a more elegant and system-efficient solution than a bolt-on dedicated vibration control damper. However, the proposed framework requires significant cooperation between different design teams, necessitating technological innovations and organizational changes.

The active control method offers greater adaptability and performance potential, but it also introduces complexity, reliability issues, and increased power requirements that must be carefully considered in blade design.

### Hybrid control

Hybrid control schemes combine passive and active control methods. They offer the advantages of active control systems by adapting to varying loading patterns and structural responses, while consuming less energy. Sonkusare and Bera (2025) developed a hybrid vibration control system that combines negative stiffness, tuned mass dampers (TMDs), and inerters to reduce the edgewise (in-plane) vibrations of wind turbine blades, achieving a 14–36% reduction in displacement and force peaks compared to traditional TMDs. However, this approach requires precise tuning and is challenging to implement due to limitations in blade rotation and internal space. The research works of Chen et al. (2024) integrated adaptive dual-layer sliding mode maximum power point tracking (MPPT) torque control with feedback and feedforward (FBFF) blade pitch control to analyze the wave-induced resonance in offshore wind turbines. A noticeable reduction in root moments at the blade and tower vibration was observed in the simulated outcomes. The dependency upon precise environmental data limits the scheme’s application.

## Future directions

Aeroelasticity in wind turbine blades is rapidly becoming one of the most exciting research fields, with several new avenues of research emerging. Advanced structural dynamics models combined with CFD are becoming popular modeling techniques, allowing for more accurate predictions of blade aeroelastic behavior. The key to the future is in developing reduced-order models based on CFD and FEA that can capture geometric nonlinearities, dynamic stall effects at high angles of attack, and nonlinear coupling due to fluid-structure interaction (especially for FOWTs). However, the computation cost still poses a significant challenge. This led to the creation of more efficient methods, like the nonlinear frequency-domain analysis, which helps reduce computation time without sacrificing accuracy.

Machine learning and artificial intelligence (AI) methods may offer promising opportunities for advancing aeroelastic prediction and control. These techniques could pave the way for real-time instability prediction and help develop adaptive control strategies that optimize turbine performance even in extreme operating conditions. Passive dampers are usually incapable of suppressing various blade resonance types in harsh offshore environments. Technical research is rapidly advancing in adaptive and semi-active control systems, with a focus on internal and primary resonance.

The advancements in composite materials, innovative structures, and passive damping technologies provide new opportunities for mitigating instability and resonance. Further, functionally graded materials and bio-inspired designs may pave the way for thrilling future exploration. Embedded piezoelectric patches or shape memory alloys within the blade laminate can act as sensors and actuators, enabling distributed energy harvesting from blade vibrations that simultaneously suppresses resonance and aerodynamic instabilities.

Since the size of wind turbines has grown significantly over the years, FOWTs pose additional aeroelastic challenges due to coupling between various blade modes and platform motions. The future of managing internal and primary resonances, as well as dynamic stalls, in large offshore blades lies in adopting a comprehensive, intelligent, and adaptive approach. Further advanced research, combining nonlinear modeling, AI-powered digital twins, cutting-edge materials and structures, and targeted active control, will ensure that the next generation of FOWTs remains structurally integral and economically feasible.

## Conclusions

The advancement of the wind energy sector demands larger wind turbines to achieve higher rated power and enhanced efficiency. It is therefore essential to ensure that the potential resonance frequencies of a wind turbine blade are different from its natural frequencies when it is fabricated. The primary excitation frequencies are avoiding the rotor speed (1 Ω) and its harmonics (2 Ω, 3 Ω…). It is essential to avoid excitation frequencies and the coincidence of natural frequencies. Several researchers have treated rotating wind turbine blades as cantilever beams to study their oscillations. The Euler-Bernoulli beam theory has been applied extensively because it is simpler than other beam models. Many scholars have developed nonlinear dynamic models for wind turbine blades undergoing bending in both flapwise and edgewise motions, without considering coupling effects; however, few have included geometric nonlinearities. The parameters, including varying slenderness ratios, aspect ratios, twist angles, hub radii, rotor speeds, and inertias, were observed to influence the natural frequencies of the blade modes. During the initial design stage, the Euler-Bernoulli beam model proved to be a powerful tool in structural dynamics modeling due to its simplicity and computational efficiency. The first mode natural frequencies calculated by various beam models shows a good agreement. However, the second flapwise frequencies computed using the Euler-Bernoulli model differ by approximately 5.23%, those using the Timoshenko model by 3.13%, and those through the Rayleigh model by 3.4% from the geometrically exact beam formulations.

The edgewise response, flutter speed, and occurrence of primary resonance of an NREL 5-MW blade with coupled modes are calculated in this work as an example to demonstrate the usefulness of the Euler-Bernoulli model. Primary, subharmonic, and superharmonic resonances were observed due to various nonlinearities in the system. Analytical responses were derived using the harmonic balance and multiple-scale methods commonly employed in existing literature for these resonance phenomena. Steady-state responses during internal and primary resonances have been analyzed through frequency response curves and phase diagrams. The equations of motion mentioned in most previous works were partial differential equations, and it is usually hard to find closed-form solutions.

Classical flutter has not been spotted in commercial wind turbines; however, it remains a possibility for future designs of larger blades. Researchers are actively analyzing the flutter gap (gap between flutter speed and operating speed) as the blade size increases. Theodorsen’s model has been used widely to examine the influence of bend-twist coupling on flutter speed. Although some recent studies have employed advanced CFD methods to model the unsteady aerodynamic behavior, these models have faced challenges due to higher computational costs and complexity. However, Euler-Bernoulli beam models vastly overestimated flutter speeds compared to the geometrically exact beam model for NREL 5-MW wind turbine blades.

Negative damping of the flapwise and edgewise motions may occur, resulting in so-called SIV, which has been a known concern for turbine blades. SIV poses a potential risk of growth and significantly contributes to the fatigue loading experienced by the blades, ultimately reducing their operational lifespan. Investigators are developing new tools for calculating the aerodynamic damping of wind turbines under various operating conditions. Smart rotor designs are also under development to reduce the aerodynamic load fluctuations and increase the damping of the blade modes. It was observed that adjusting the mode shapes of wind turbines has the potential to moderate SIV. The lengthy, flexible NREL 5-MW and IEA 15-MW blades are sensitive to flutter instabilities, where aerodynamic damping can decrease significantly at certain operational speeds.

Several outstanding issues require further investigation:

• The flapwise and edgewise vibrations have been examined mainly independently, rather than as a coupled system. This gap should be addressed by focusing on the first edge mode, the first two flap modes, and their coupling.
• Many scholarly articles have neglected the torsion mode when analyzing coupled-mode systems. They have focused solely on deriving the basic equations for the coupled edgewise, flapwise, and torsion vibrations.
• Many focused solely on the fundamental, primary, subharmonic, and superharmonic resonances, with less emphasis on internal and combination resonances. An internal resonance involves all four basic modes; however, a combination resonance occurs when internal and primary resonances co-occur, which is necessary to understand the mechanisms of energy transfer among the various modes.
• Numerous deficiencies remain in the effective strategies for mitigating primary resonance through combination resonance.
• Aeroelastic instability models have primarily been used for incompressible flows in flutter investigations. However, the relative velocities are in the subsonic region when large wind turbines operate under flutter conditions. CFD-based methods may be employed to account for compressibility effects precisely.
• The unsteady BEM methods demand considerably less computational resources than CFD models. BEM enables the integration of dynamic wake and stall models, making it a more effective alternative that can be utilized in conjunction with one-dimensional beam models.
• Further investigations are needed to determine the decrease in flutter limits resulting from moving the centroid towards the leading or trailing edge.
• The accuracy of the impact of inflow conditions on SIV, as examined through cost-effective substitutes to CFD simulations—specifically surrogate models—requires further research.

It is essential to distinguish between aeroelastic instabilities and resonance, and to prevent both for future (long slender blade) designs. This study’s outcomes provided valuable insights into the nonlinear dynamics of wind turbine blades, particularly aeroelastic instabilities and resonance. Furthermore, it highlighted the research gaps in the current literature, paving the way for future investigations to reduce the vibrations experienced by wind turbine blades. In the future, extensive full-scale field experiments will be required to test high-fidelity CFD wind turbine models operating in real-world environments.

## Data Availability

Data will be made available upon reasonable request.*
